# Impact of the SARS-CoV-2 Epidemic on Lung Cancer Surgery in France: A Nationwide Study

**DOI:** 10.3390/cancers13246277

**Published:** 2021-12-14

**Authors:** Pierre-Benoit Pages, Jonathan Cottenet, Philippe Bonniaud, Pascale Tubert-Bitter, Lionel Piroth, Jacques Cadranel, Alain Bernard, Catherine Quantin

**Affiliations:** 1 Department of Thoracic Surgery, Centre Hospitalier Universitaire Dijon, Bocage Central, 21079 Dijon, France; pierrebenoit.pages@chu-dijon.fr (P.-B.P.); alain.bernard@chu-dijon.fr (A.B.); 2 INSERM UMR 1231, Centre Hospitalier Universitaire Bocage, University of Burgundy, 21079 Dijon, France; 3 Biostatistics and Bioinformatics (DIM), Dijon University Hospital, University of Burgundy Franche-Comté, BP 77908, 21079 Dijon, France; jonathan.cottenet@chu-dijon.fr; 4 Faculty of Medicine, University of Bourgogne-Franche-Comté, 21000 Dijon, France; philippe.bonniaud@chu-dijon.fr (P.B.); lionel.piroth@chu-dijon.fr (L.P.); 5 Reference Center for Rare Pulmonary Diseases, Pulmonary Medicine and Intensive Care Unit Department, Dijon University Hospital, BP 77908, 21079 Dijon, France; 6 High-Dimensional Biostatistics for Drug Safety and Genomics, Paris-Saclay University, UVSQ, Inserm, CESP, 94800 Villejuif, France; pascale.tubert@inserm.fr; 7 Clinical Investigation Center, Clinical Epidemiology/Clinical Trials Unit, Dijon University Hospital, 21079 Dijon, France; 8 Infectious Diseases Department, Dijon University Hospital, BP 77908, 21079 Dijon, France; 9 Chest Department and Constitutive Center for Rare Pulmonary Disease, Hôpital Tenon, AP-HP, Inflammation-Immunopathology-Biotherapy Department (DHU i2B) and Sorbonne University, 75020 Paris, France; jacques.cadranel@aphp.fr

**Keywords:** SARS-CoV-2, lung cancer, mortality, administrative data, hospital, surgical activity volume

## Abstract

**Simple Summary:**

Few studies have investigated the link between SARS-CoV-2 and health restrictions and its effects on the health of lung cancer (LC) patients. This study aimed to assess the impact of SARS-CoV-2 on activity volume, postoperative complications and in-hospital mortality (IHM) for LC resections in 2020 at the national level in France. Our study shows a decrease in the volume of LC resections, especially during the first lockdown. We also show that only 0.43% of patients hospitalized for LC surgery during 2020 developed a SARS-CoV-2 infection, but this low rate is counterbalanced by a high IHM (21%) in these 51 patients. Our findings suggest that, even if the IHM is high, LC surgery is feasible during a pandemic provided that the general guidance protocols edited by the surgical societies are respected. Therefore, this study provides further arguments to encourage teams to test for COVID-19 prior to surgery and patients to be vaccinated.

**Abstract:**

Few studies have investigated the link between SARS-CoV-2 and health restrictions and its effects on the health of lung cancer (LC) patients. The aim of this study was to assess the impact of the SARS-CoV-2 epidemic on surgical activity volume, postoperative complications and in-hospital mortality (IHM) for LC resections in France. All data for adult patients who underwent pulmonary resection for LC in France in 2020, collected from the national administrative database, were compared to 2018–2019. The effect of SARS-CoV-2 on the risk of IHM and severe complications within 30 days among LC surgery patients was examined using a logistic regression analysis adjusted for age, sex, comorbidities and type of resection. There was a slight decrease in the volume of LC resections in 2020 (*n* = 11,634), as compared to 2018 (*n* = 12,153) and 2019 (*n* = 12,227), with a noticeable decrease in April 2020 (the peak of the first wave of epidemic in France). We found that SARS-CoV-2 (0.43% of 2020 resections) was associated with IHM and severe complications, with, respectively, a sevenfold (aOR = 7.17 (3.30–15.55)) and almost a fivefold (aOR = 4.76 (2.31–9.80)) increase in risk. Our study suggests that LC surgery is feasible even during a pandemic, provided that general guidance protocols edited by the surgical societies are respected.

## 1. Introduction

With an estimated 2.2 million new cases in 2020 and 1.8 million deaths, lung cancer (LC) remains the leading cause of death by cancer worldwide [1]. For early stage and locally advanced LC, surgical resection remains the standard of care that provides the highest overall survival [2]. SARS-CoV-2 disease 2019 (SARS-CoV-2), emerged as a new infectious disease at the end of 2019 and spread quickly worldwide. Lockdowns and restrictions policies of various temporal and geographical intensities were implemented all around the world in order to control the pandemic.

In France, the first SARS-CoV-2 wave started on 23 February 2020, leading to an initial two-month lockdown (from 17 March to 11 May 2020, with an epidemic peak in mid-April 2020). A second and less stringent one-month lockdown was implemented from 28 October to 28 November 2020. Despite these measures, from the 1st of March to the 31th of December 2020, around 260,000 patients were hospitalized for SARS-CoV-2, and 65,000 of these patients died from the infection [3].

Whilst SARS-CoV-2 induced minimal illness in most of the patients, a significant proportion of patients required management in intensive care, which stretched the health care system and forced French hospitals to double their intensive care capacity [3]. The organization of care and care practitioners had to be altered to meet the unexpected demands, leading to a reduction in all programmed hospitalizations in non-SARS-CoV-2 patients, especially for surgical interventions [4]. In this context, delaying surgical resection for LC was an ethical problem because such delays have the real potential to worsen survival outcomes [3].

The aim of this study was to assess the impact of the SARS-CoV-2 epidemic in 2020 on surgical activity volume, postoperative complications and in-hospital mortality for LC resections in France using the French national administrative database.

## 2. Materials and Methods

### 2.1. Study Design and Participants

We conducted a retrospective cohort study using the national database (Programme de médicalisation des ystems d’information, PMSI), which is designed to include discharge summaries for all inpatients admitted to public and private hospitals in France [5]. The information in these abstracts is anonymous and covers both medical and administrative data. Diagnoses identified during the hospital stay are coded according to the 10th edition of the International Classification of Diseases (ICD-10), and procedures performed during the hospitalization are coded according to the French Common Classification of Medical Procedures.

This study was approved by the Comité éthique et scientifique pour les recherches, les études et les évaluations dans le domaine de la santé (CESREES, ethics and scientific committee for research, Studies and evaluation in health, 9 June 2020) and the Institut des données de santé (INDS, French institute of health data, registration number 1611357, 15 June 2020) and authorized by the Commission nationale de l’informatique et des libertés (CNIL, French data protection authority, registration number DR-2020–250, 3 July 2020). Written consent was not needed for this study as it was a retrospective study and the national data used were anonymous.

All data for adult patients (18 years and older) who underwent pulmonary resection for LC in France from January 2018 to December 2020 were collected from the national administrative database. We selected patients with both a diagnosis of primary LC, coded as the principal discharge diagnosis (ICD-10 code C34) [6,7], and a procedure of pulmonary resection during the same stay. Only the first stay was taken into account and the patient was therefore counted only once.

The number of patients with LC resection and the frequency of patients who underwent pulmonary resection among all patients hospitalized with LC were estimated by month for 2020 and compared graphically to the two previous years (2018 and 2019).

We also estimated the risk of developing SARS-CoV-2 infection, and the risk of severe complications and in-hospital mortality in infected patients, within 30 days after surgery.

### 2.2. Patient Characteristics

Baseline demographics included age and sex. From the national administrative database, we included the following comorbidities: pulmonary disease, heart disease, peripheral vascular disease, liver disease, neurological diseases, endocrine disease, metabolic disease, renal disease, infectious disease, haematological disease, other malignant disease and LC relative/associated therapies. We also calculated a modified Charlson Comorbidity Index as a marker of comorbidity.

SARS-CoV-2 patients diagnosed during the surgery stay were identified by their primary diagnoses, related diagnoses or associated diagnoses using the ICD-10 codes U0710, U0711, U0712, U0714 or U0715. Thus, we identified all patients who presented a SARS-CoV-2 infection during hospitalization for surgery.

Finally, the type of LC resection (sublobar resection, lobectomy, bilobectomy and pneumonectomy) was identified.

Transfer to an intensive care unit (ICU) was considered during the surgery stay or within the first 30 days after the operation.

Postoperative complications were retrieved: pneumonia, acute respiratory distress syndrome (ARDS), bleeding, respiratory failure, heart failure, acute renal failure, infectious complications, phlebitis and pulmonary embolism.

### 2.3. Outcome Measurements

In-hospital mortality (IHM) was defined as any patient who died in hospital during the surgery stay or within the first 30 days after the operation.

As mentioned above, severe complication was defined as any patient who developed at least one of the following complications during the surgery stay or within the first 30 days after the operation: pneumonia, ARDS, respiratory failure, heart failure, acute renal failure, infectious complications or pulmonary embolism.

We also considered severe complications based on the Clavien Dindo classification [8], which means that the previous complications were classified as grade III and IV in the classification (admission to ICU for more than 48 h, surgical revision or dialysis).

### 2.4. Statistical Analysis

The annual and monthly number of LC patients hospitalized for a pulmonary resection were determined for each of the three years. We also calculated the monthly frequency of patients who underwent pulmonary resection among all patients hospitalized with LC. These statistics were also calculated by type of pulmonary resection.

We described the patient characteristics and complications for all patients who underwent LC resection. Qualitative variables were summarized as frequencies (percentages), and quantitative variables were summarized as means (standard deviation (SD)) and medians (interquartile range (Q1–Q3)). Variables were compared between the three years using the Chi-2 test or the Fisher’s exact test for qualitative variables, and the Student’s *t*-test or Kruskall–Wallis test was used to compare quantitative variables.

The above-mentioned descriptive statistics were also calculated for SARS-CoV-2 and non-SARS-CoV-2 patients in 2020 and compared to 2019 (reference year) using the above-mentioned statistical tests, as appropriate, after looking at the stability of the figures for 2018 and 2019.

The effect of SARS-CoV-2 on the risk of IHM and severe complications among LC surgery patients was analysed using logistic regression adjusting for age, sex, comorbidities and type of LC resection. We first considered the year 2019 (before the pandemic) as a reference to study both the effect of SARS-CoV-2 infection and the changes in practice and supply of care due to constraints on the health care system. We then restricted this analysis to the year 2020. The variables included in the multivariate models were those significant in univariate models with a *p*-value < 0.20. Correlations were studied and interactions tested. The results were reported as crude and adjusted odds ratios (OR) and 95% confidence intervals (CIs).

The statistical significance threshold was set at <0.05. All analyses were performed using SAS (SAS Institute Inc, Version 9.4, Cary, NC, USA).

## 3. Results

**Figure 1:**
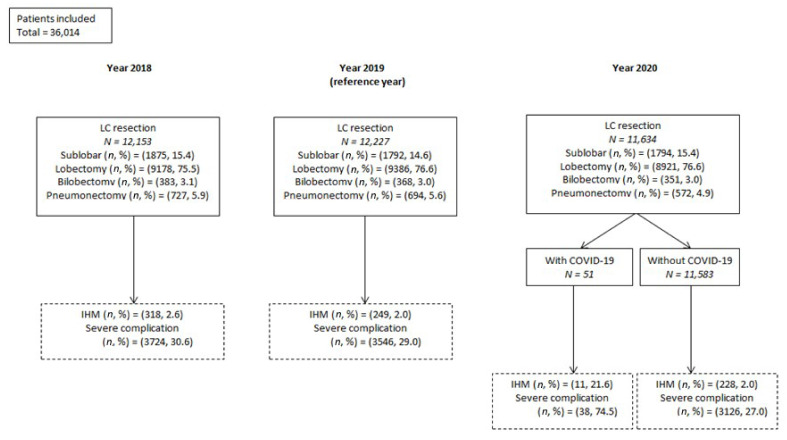
Study population flow chart by year of inclusion.

From the 1st of January 2018 to the 31st of December 2020, 36,014 patients were operated on for LC in France (Figure 1), of which about 15% underwent sublobar resection (8.5% of wedge resections and 6.5% of segmentectomies), 76% lobectomy, 3% bilobectomy and 5% pneumonectomy.

### 3.1. Surgical Activity Volume

**Figure 2:**
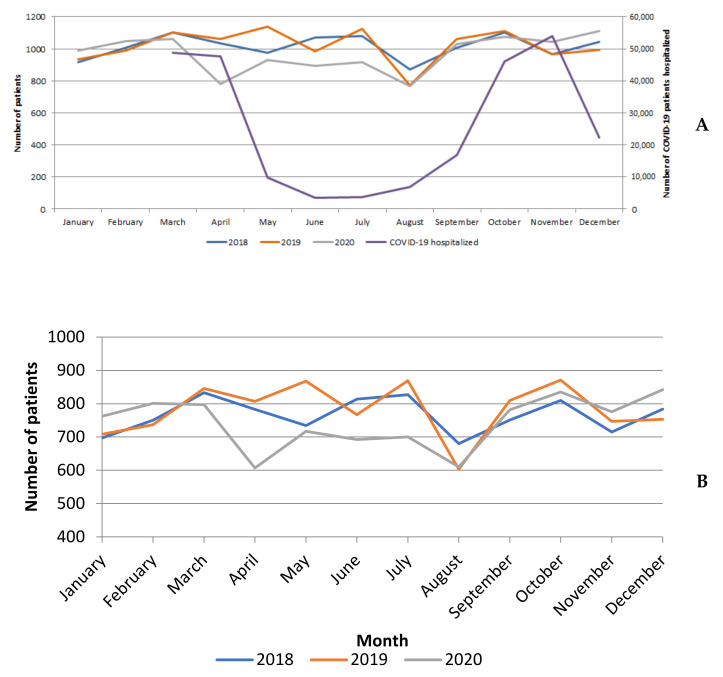

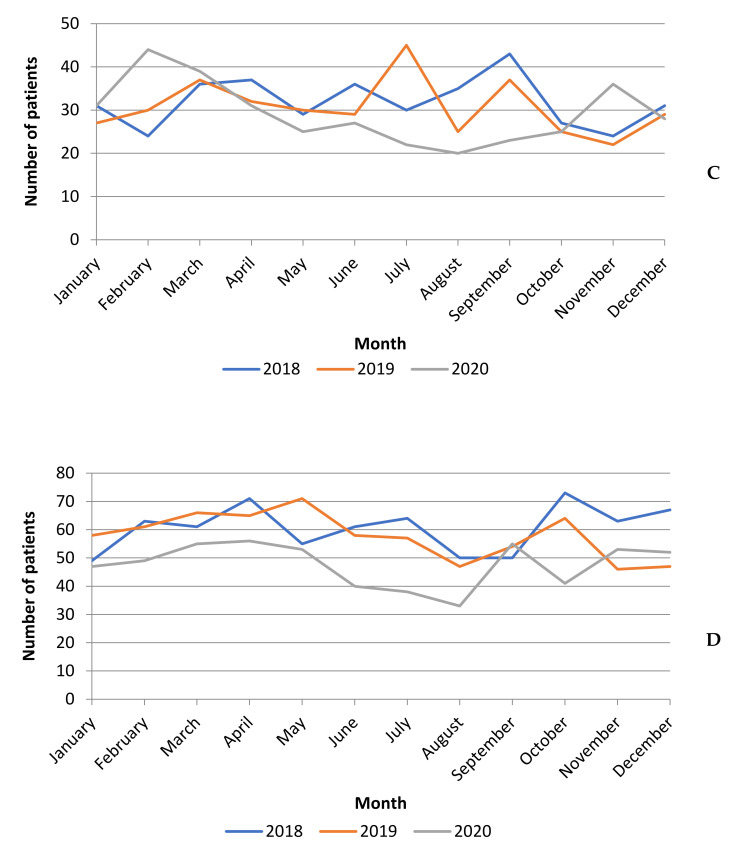

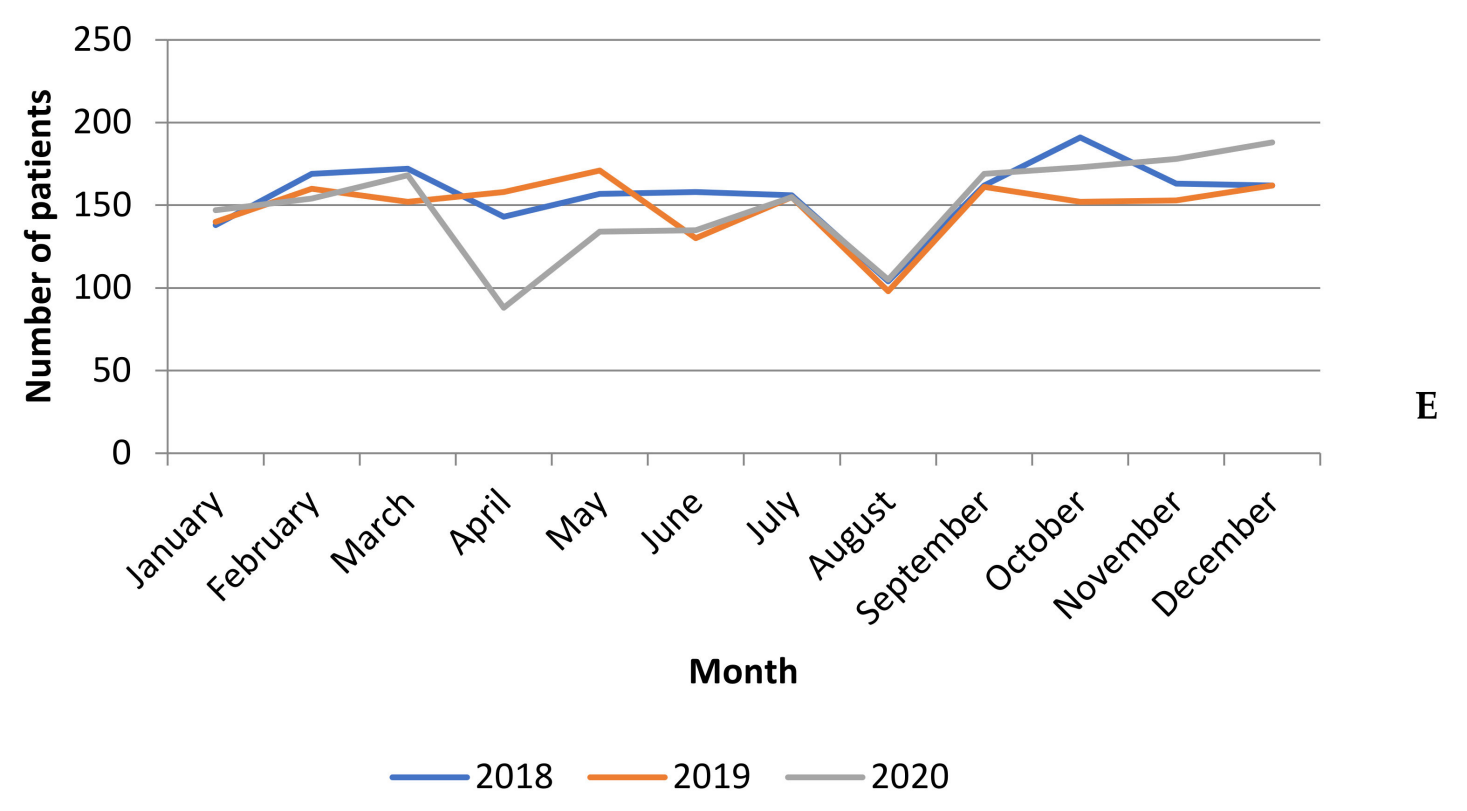
Number of patients with lung cancer resection by month in 2018, 2019 and 2020, (**A**) overall and by type of surgery; (**B**) lobectomy; (**C**) bilobectomy; (**D**) pneumonectomy; (**E**) sublobar.

There was a slight decrease in the volume of LC resections in 2020 (*n* = 11,634), as compared to 2018 (*n* = 12,153) and 2019 (*n* = 12,227), with a noticeable decrease in April 2020 (the peak of the first wave of epidemic in France). Thereafter, the activity volume remained lower than the corresponding levels in 2018 and 2019 until August 2020 (Figure 2A). Then, the activity volume increased regularly until the end of 2020, with the last 2 months exceeding the corresponding levels of 2018 and 2019, resulting from an increase in the volume of lobectomies (Figure 2A,B).

The number of bilobectomies and pneumonectomies decreased gradually from March to July and then returned to the level of 2018 and 2019 (Figure 2C,D).

Regarding the monthly frequency of pulmonary resections among patients hospitalized with LC, we observed a lower frequency than in previous years from March (beginning of the first wave and initial lockdown in France) to July (Figure S1), irrespective of the type of LC resection (Figure S2).

### 3.2. Patient Characteristics

#### 3.2.1. Whole Sample of Patients

Patient characteristics are reported in Table 1. The mean age (≈66 years) and sex ratio (≈1.5) were fairly stable over the years.

As compared to the two previous years, patients in 2020 were more frequently women and had a Charlson score ≥ 3 more frequently; they had significantly less history of pulmonary disease, malignant lesions and previous therapies, particularly in relation to 2018 (Table 1).

#### 3.2.2. LC Resections in Patients with SARS-CoV-2 Infection

In 2020, 51 patients who had LC resections were diagnosed with SARS-CoV-2 infection (0.43%) during their hospitalization (Table 1). These 51 patients were all patients who presented a SARS-CoV-2 infection during their hospitalization for surgery. As compared to non-SARS-CoV-2 2019 patients (*n* = 12,227), the patients in 2020 infected by SARS-CoV-2 were generally in worse health. They had a significantly higher occurrence of past histories of pulmonary diseases (72.6% vs. 31.6%, *p* < 0.0001) and metabolic diseases (29.4% vs. 13.7%, *p* = 0.0012). They also tended to have a higher occurrence of neurological disorders (9.8% vs. 4.3%, *p* = 0.056), and endocrine diseases (21.6% vs. 12.6%, *p* = 0.0557), and a higher occurrence of a Charlson score ≥ 1 (Table 1).

It should be noted that none of the comorbidity frequencies were significantly different between 2019 and 2020 in non-SARS-CoV-2 patients.

### 3.3. Thirty-Day in-Hospital Mortality and Postoperative Complications

#### 3.3.1. Whole Sample of Patients

Compared to 2018 and 2019, the patients operated for LC in 2020 had significantly less respiratory failure, and fewer infectious complications and severe complications (Table 2). The frequencies of complications and IHM were different between the 3 years due in particular to higher rates in 2018 (Table 2).

#### 3.3.2. LC Resections in Patients without SARS-CoV-2 Infection

For patients in 2020 without SARS-CoV-2 infection, the frequencies of respiratory failure, sepsis and severe complications were significantly lower than for patients in 2019, while the frequency of transfer to the ICU was significantly higher.

#### 3.3.3. LC Resections in Patients with SARS-CoV-2 Infection

Compared to 2019, patients in 2020 with SARS-CoV-2 were more likely to develop pneumonia (60.8% vs. 15.4%, *p* < 0.0001), ARDS (29.4% vs. 6%, *p* < 0.0001), respiratory failure (49% vs. 12.3%, *p* < 0.0001), acute renal failure (17.7% vs. 5.1%, *p* = 0.001), infectious complications (29.4% vs. 11.6%, *p* < 0.0001), and severe complications (74.5% vs. 29%, *p* < 0.0001).

As compared to patients in 2019, SARS-CoV-2 patients were dramatically more likely to be transferred to the ICU (58.8% vs. 22.0%, *p* < 0.0001), and IHM was ten times higher (21.6% vs. 2.0%).

### 3.4. Factors Associated with 30-Day in-Hospital Mortality and Severe Complications in Patients with SARS-CoV-2 Infection

When considering patients operated for LC in 2019 as a reference, after adjustment for age, sex and comorbidities, the main predictive factors of severe postoperative complications were: SARS-CoV-2 infection (aOR = 4.75 (2.31–9.80)), bilobectomy (aOR = 1.92 (1.54–2.38)), pneumonectomy (aOR = 2.14 (1.80–2.54)), history of pulmonary disease (aOR = 5.91 (5.50–6.34)), and history of infectious disease (aOR = 16.67 (9.05–30.70)) (Table 3). We found similar results when considering severe complications based on the Clavien Dindo classification (Table S1).

Again, with 2019 patients operated for LC as a reference, the main predictive factors of IHM were: SARS-CoV-2 infection (aOR = 7.16(3.30–15.52)), bilobectomy (aOR = 2.35 (1.48–3.74)), pneumonectomy (aOR = 2.94 (1.99–4.32)), history of pulmonary disease (aOR = 4.09 (3.25–5.13)), liver disease (aOR = 4.99 (2.95–8.44)), renal disease (aOR = 2.58 (1.82–3.66)), and history of infectious disease (aOR = 5.12 (3.08–8.53)) (Table 3).

Restricting to the year 2020 and considering the same adjustment factors, the risk of IHM associated with SARS-CoV-2 infection was almost seven times higher compared with patients without SARS-CoV-2 infection, and the risk of severe complications was five times higher (Table S2).

Thus, the risk of IHM or severe complications were consistent, whether the reference non-SARS-CoV-2 group was from 2019 or 2020.

## 4. Discussion

In 2020, we observed a slight drop in hospitalizations for lung cancer surgery during the first lockdown, with a return to almost normal activity (from August onwards), but surgical activity does not seem to have been impacted by the second wave nor by the second lockdown. There was a slight increase at the end of 2020 as compared to 2019, probably to catch up with the activity that could not be performed before. In particular, our study shows that the surgical activity volume for all LC surgeries decreased dramatically from March to April in France. The SARS-CoV-2 pandemic started in France in February 2020, leading to an initial two-month lockdown (from 17 March to 11 May 2020, with an epidemic peak in mid-April 2020). Because of the large number of cases, the health system was quickly saturated. This decrease in surgical activity volume may be due to several factors. On the one hand, a new organisation was set up to increase the capacity of the ICU in order to deal with COVID-19 hospitalizations, with the deployment of the anaesthesiology and paramedical personnel from the operating room to the ICU. At the same time, there were inadequate data about the risk of patients becoming infected with SARS-CoV-2. Hence, on 17 March 2020, the French Society of Thoracic and Cardiovascular Surgery sent a message to all surgeons indicating that they should postpone all cardiac and thoracic surgeries, except for emergency surgery. Early French guidelines (from the French High Council of Public Health) recommended discussing each case in a multidisciplinary board meeting for non-small cell lung cancer and to consider alternative treatments [9]. The first recommendations for organizing access to care for patients whose procedure could not be postponed were published at the end of April 2020 and updated two months later by the French Society of Anaesthesia and Intensive Care. They recommended that each patient be surveyed for symptoms of SARS-CoV-2 infection, or contact with a person with COVID-19 within 15 days prior to surgery. As soon as polymerase chain reaction (PCR) testing was widespread, it was systematically requested in the 72 h before surgery. In case of positive preoperative PCR testing, it was recommended that the surgery be delayed for 2 or 3 weeks or until the patient was asymptomatic. After, the isolation period, all patients should have been tested. If the surgery could not be conducted, the ESMO guidelines recommended radiation for early stage LC [10].

For LC surgery, there was a dilemma [11,12] between the risk of contracting SARS-CoV-2 and the risk of delaying LC surgery, both of which have been reported to impact survival [13]. Indeed, patients suffering from LC have a higher risk of developing SARS-CoV-2 infection and are also significantly more likely to develop severe complications, especially ARDS, leading to a high mortality rate (8% to 30%) [14,15].

In this study, we found a decrease in the number of major surgeries, such as bilobectomy and pneumonectomy, which could have several explanations. Firstly, patients who undergo LC resection require close monitoring in the ICU, where beds were heavily occupied by SARS-CoV-2 patients during much of 2020. Secondly, some guidelines published during the pandemic recommended prioritizing radiation [16] for locally advanced LC, thereby limiting the use of resection. Thirdly, the disease may have progressed due to COVID-19-related delays and become unresectable in some cases [17].

Although there was a dramatic decrease in surgical activity volume in April, it increased regularly after August 2020, particularly due to the increase in the number of lobectomies. The increase was impacted by the progressive decrease in number of SARS-CoV-2 ICU hospitalizations after April 2020 and continued until the end of the year, despite the second lockdown from 28 October to 28 November 2020. In fact, during the second wave, hospitals were better organised, there was an increased number of ICU beds in France, and surgical pathways had been set up to ensure the maintenance of surgical management for LC. However, even if the activity volume for LC surgery increased after August, the yearly volume never recovered to the volume of the previous year, suggesting that a number of patients never underwent surgery.

Fifty-one patients operated for LC were diagnosed with SARS-CoV-2 infection during their hospitalization. It is possible that the infection was not detected before hospitalization or that it was acquired in-hospital [18,19,20]. No data were available to know if the infection started before the hospitalization or after the surgery: hospital discharge abstracts provide all diagnoses recorded for a given stay, but not their exact chronology. These patients were likely to be in poor general condition, mainly due to a history of pulmonary disease and diabetes; such patients have already been reported to be at risk of developing SARS-CoV-2 infection [21,22]. We found that, when compared to patients from 2019, the patients from 2020 who were infected with SARS-CoV-2 were generally in poorer health.

Regarding the 30-day IHM and postoperative complications, we found that non-SARS-CoV-2 patients had a low IHM (around 2%) and a low rate of postoperative complications. Over the past years, there has been a trend of decreasing IHM after LC surgery. Using the French administrative database, Morgant et al. reported an IHM after LC surgery of 3.5% in 2005, and 2.5% in 2012 for all types of pulmonary resection [23]. This trend was also reported by Broderick et al. in the Society of Thoracic Surgeons (STS) clinical database in the US, with an IHM of 1.3% in 2017 [24]. The lower IHM in the STS database study could be linked to a larger proportion of sublobar pulmonary resection (around 23%, divided into 15.4% wedge and 6.9% segmentectomy) when compared to our study (15% sublobar resection). Postoperative complications tended to decrease during our study period, but remained higher than their corresponding values in the STS database. Specifically, Broderick et al. [24] reported a lower postoperative incidence of pneumonia, respiratory failure and ARDS in the US than what was observed in our study. These differences could be explained in several ways. Firstly, there is a higher proportion of sublobar resection in the US study, which is reported to induce less postoperative complications. Secondly, clinical databases (such as the STS database) are known to underestimate postoperative complications. Thirdly, the STS database included less than 50% of LC resection in the US, whereas our study, which is based on an administrative database, included all LC resections performed in France during the study period [24].

SARS-CoV-2 patients in our study had a high rate of postoperative complications, especially respiratory complications and acute renal failure. The incidence of pneumonia in SARS-CoV-2 patients was four times higher (around 60%) than in the rest of our cohort, which led to a high incidence of ARDS (around 30%) and the need for ICU transfer in 58% of cases. As previously described, patients who develop a SARS-CoV-2 infection have a high risk of developing pulmonary complications [25]—this includes patients diagnosed with LC [26,27]. In a recent (2021) national study in France, Bernard et al. reported a high proportion of acute respiratory failure (29.2%) in case of SARS-CoV-2 infection [28]. Another report confirmed the high rate of transfer to the ICU in LC patients who develop respiratory failure [15].

In Bernard et al., the IHM in LC patients with a SARS-CoV-2 infection was around 25% [24]. In our cohort, 21.6% of the patients with this infection died after LC surgery. Regarding the impact of SARS-CoV-2 infection on LC surgery outcomes, three smaller studies have reported an IHM ranging from 27% to 43% [29,30]. However, as stated by Seitlinger et al. [12], some of the conclusions of these studies may seem hasty because the small sample sizes make it difficult to distinguish between the effect of SARS-CoV-2 and that of the main comorbidities.

We found that only 0.43% of patients who received LC resection during the study period developed a SARS-CoV-2 infection. This percentage may appear low in view of what has been published in the literature [18,19,31]. However, most studies focused on patients hospitalized in specific medical departments whereas we included all surgery patients. The care of these two types of patients cannot be compared. Two studies including about 100 patients reported a confirmed SARS-CoV-2 infection in the perioperative period among about 5% of patients who received LC resection or thoracic surgery [31,32]. The percentage observed in our study is still much lower than what has been observed in comparable patients undergoing surgery for LC. Moreover, other studies conducted to measure the impact of hospital-acquired infections among patients hospitalized with SARS-CoV-2 have found that up to 12.5% of SARS-CoV-2 infections are acquired in hospital [20]. It thus seems that the percentage of hospital-acquired infections in our series of around 12,000 patients is very low, which tends to confirm the effectiveness of the measures taken to ensure the safety of these patients, in particular carrying out a PCR test on hospital admission. Several other publications studying LC surgery in the same period reported no infection, but these were performed in very small series [33,34]. In the literature, we found few results regarding SARS-CoV-2 infection during hospitalization for LC surgery, but we are also well aware that surgical inpatients were highly selected to ensure that they were free of COVID-19. It also seems inappropriate to compare patients who were operated on with those who did not undergo surgery since these unoperated patients, who are often metastatic, frequently return to hospital. They are also mostly treated with chemotherapy and chemo-immunotherapy, which puts them at a greater risk of COVID-19 infection and hospitalization. Since the hospitalized patients were stringently selected for surgery, we supposed that the frequency should be about that of the general population. We thus retrieved the overall national incidence of SARS-CoV-2 using official figures of the French government regarding incidence of COVID-19, starting in May 2020 (available on https://www.data.gouv.fr/fr/datasets, accessed on 30 September 2021)—there are no available official data before May 2020). The rate of COVID-19 in the French general population varies from 12 to 1348 per 100,000 persons between May 2020 to December 2020 with a peak in October, and an overall rate of 430 per 100,000 persons for 2020 (May–December). It seems reasonable to assume that the incidence of COVID-19 infection in our population of patients hospitalized for LC surgery (0.43% or 430 per 100,000) is very close to the incidence observed in the general population in the same period.

However, our low rate of infection is balanced by a very high rate of IHM (around 21%) in the infected group. This finding is consistent with a study on nosocomial SARS-CoV-2 in a “green ward” in England, which reported an IHM of 21% among patients who stayed more than 7 days and contracted SARS-CoV-2 during the stay [18]. In our study, the high IHM rate may not be so surprising, to the extent that the patients from 2020 infected with SARS-CoV-2 generally had poorer health than patients from 2019. However, we found that SARS-CoV-2 infection was still associated with IHM, since the risk increased nearly sevenfold, and with severe complications, with a fivefold increase, after adjusting for age, sex, comorbidities and type of LC resection. It should be taken into account when deciding whether or not to postpone the surgical procedure. Indeed, delaying the surgery has been reported to have negative impact on survival [35,36,37], and allowing the LC to progress means having to provide more costly surgery and/or chemotherapy [38]. Recent guidelines from the International Association for the Study of Lung Cancer (IASLC) recommend making decisions about the type of treatment and the timing of its administration by considering the stage of the tumour [39,40] and the capacity of the health care system [11].

The main strength of this study is that it uses nationwide data covering the whole year 2020, which included the first two waves of the COVID-19 pandemic and associated lockdowns, and distinguishes SARS-CoV-2 vs. non-SARS-CoV-2 LC patients on a monthly basis, providing a comparison with the two previous years. This work is unique for the large number of patients included and the comprehensiveness of the data. In France, the national hospital database includes information from all private and public French hospitals; it is used for the allocation of hospital budgets and encourages high levels of data coherence, accuracy and exhaustiveness. The quality and completeness of the coding are evaluated locally by the medical information departments of the individual institutions and nationally by the national health insurance system. Due to the reliability and quality of this database we have been able to use this database in several papers published in international journals of high rank on several topics [25,41], in particular on the same subject [6,7,42,43,44].

One of the limitations could be the potential for biases related to misclassification or under-detection, especially for comorbidities. Coding practices vary significantly among institutions. Nevertheless, coding quality is checked by medical information professionals in each hospital to correct diagnoses and increase the level of recorded comorbidity. In addition, we have no data on tumour stage or subsequent delay of scheduled surgery. Finally, for some variables, information may not have been completed in the discharge abstract when there was no direct impact on patient care during hospitalization (e.g., tobacco use). This is often the case for day-care but it did not apply to the hospitalizations considered in this study.

## 5. Conclusions

Our study in a large nationwide cohort of more than 36,000 patients confirms that the SARS-CoV-2 epidemic impacted surgical activity for LC in France and resulted in a large decrease in the volume of LC resections during the first lockdown. The volume only returned to typical levels towards the end of the year. After deliberation within expert groups, surgical teams made vast efforts to adapt surgical activity to the sanitary situation. This resulted in complication and hospital mortality rates that were even lower than in the previous two years, even if there is uncertainty about the nature of the patients who were not operated on.

Our study shows that only 0.43% of patients hospitalized for LC surgery developed a SARS-CoV-2 infection, suggesting that, even if the IHM is high, LC surgery is feasible in pandemic conditions provided that general guidance protocols are respected. This study provides further arguments to encourage PCR testing for SARS-CoV-2 before surgery.

## Figures and Tables

**Table 1:** Characteristics at baseline of lung cancer resection patients.

Characteristics	Years			*p*-Value (Comparison between the 3 Years)	2020 SARS-CoV-2 Patients		2020 Non-SARS-CoV-2 Patients		
	2018	2019	2020		Characteristics	*p*-Value (Comparison with 2019)	Characteristics	*p*-Value (Comparison with 2019)	*p*-Value (Comparison with 2020 SARS-CoV-2)
Hospitalized patients with lung cancer resection (*n*)	12,153	12,227	11,634		51		11,583		
Age Mean +/− std	65 +/− 10 ^‡^	66 +/− 10	66 +/− 10	0.0002	68 +/− 8	0.0615	66 +/− 10	0.8598	0.0641
Median (Q1–Q3)	66 (60–72)	67 (60–72)	67 (60–73)		70 (61–73)		67 (60–73)		
Min-Max	18–92	18–91	18–94		50–85		18–94		
Men (%)	63.4 *	62 *	60.6	<0.0001	62.8	0.9150	60.6	0.0204	0.7494
Pulmonary disease (%)	33.1 *	31.6	30.9	0.0012	72.6	<0.0001	30.8	0.1494	<0.0001
Heart disease (%)	15.7	15.5	16.3	0.1949	11.8	0.4638	16.3	0.0782	0.3798
Peripheral vascular disease (%)	9.7	9.2	9.1	0.1926	11.8	0.4668	9.1	0.8200	0.4693
Neurological disease (%)	4.2	4.1	4.3	0.6038	9.8	0.0560	4.3	0.3663	0.0678
Liver disease (%)	0.9	1	0.8	0.1705	2	0.3946	0.8	0.0643	0.3216
Renal disease (%)	2.9	2.8	3.1	0.2805	3.9	0.6523	3.1	0.1161	0.6725
Endocrine disease (%)	12.6	12.6	12.9	0.6701	21.6	0.0557	12.9	0.5711	0.0649
Metabolic disease (%)	14.1	13.7	13.8	0.6054	29.4	0.0012	13.7	0.9888	0.0012
Infectious disease (%)	0.6	0.7	0.6	0.7268	2	0.2959	0.6	0.4208	0.2654
Hematological disease (%)	5.2	5.3	5.2	0.9183	7.8	0.3506	5.2	0.6855	0.3411
Other malignant lesions (%)	43.4 *	41.7	41.8	0.0116	45.1	0.6253	41.8	0.9497	0.6295
Other therapies (%)	11.2 *	9.4	9.5	<0.0001	9.8	0.8112	9.5	0.7757	0.8131
Charlson index (%)				0.0084		0.0238		0.0013	0.0199
0	43.5	43.9	44.5		25.5		44.6		
1	24	24.9	22.8		35.3		22.8		
2	8	7.7	7.9		13.7		7.9		
≥3	24.6	23.5	24.8		25.5		24.8		

Std: standard deviation; (Q1–Q3): interquartile range; * significantly more frequent compared to 2020 (*p* < 0.05); ^‡^ significantly less frequent compared to 2020 (*p* < 0.05).

**Table 2:** Thirty-day postoperative complications and in-hospital mortality among lung cancer resection patients.

Postoperative Complications	Year			*p*-Value (Comparison between the 3 Years)	2020 SARS-CoV-2 Patients		2020 non-SARS-CoV-2 Patients		
	2018	2019	2020		Characteristics	*p*-Value (Comparison with 2019)	Characteristics	*p*-Value (Comparison with 2019)	*p*-Value (Comparison with 2020 SARS-CoV-2)
Hospitalized patients with lung cancer resection (*n*)	12,153	12,227	11,634		51		11,583		
Postoperative complications÷Pneumonia (%)	15.7 *	15.4	14.7	0.0805	60.8	<0.0001	14.5	0.0592	<0.0001
Acute respiratory distress syndrome (%)	7.7 *	6	6.2	<0.0001	29.4	<0.0001	6.1	0.8522	<0.0001
Bleeding (%)	8.7	9.6	9.5	0.0397	5.9	0.3671	9.5	0.7492	0.3803
Respiratory failure %)	13.8 *	12.3 *	11.3	<0.0001	49	<0.0001	11.1	0.0067	<0.0001
Heart failure (%)	2.6 *	2.5	2.2	0.0802	5.9	0.1414	2.2	0.0756	0.1026
Acute renal failure (%)	5.5 *	5.1	4.7	0.0146	17.7	0.0010	4.6	0.0987	0.0005
Phlebitis (%)	1.6	1.6	1.6	0.9476	3.9	0.1926	1.6	0.9193	0.1897
Pulmonary embolism (%)	1.2	1.2	1.3	0.6432	3.9	0.1341	1.3	0.5542	0.1492
Infectious complications (including sepsis) (%)	12.7 *	11.6 *	10.3	<0.0001	29.4	<0.0001	10.2	0.0005	<0.0001
Severe complications ^a^ (%)	30.6 *	29 *	27.2	<0.0001	74.5	<0.0001	27	0.0005	<0.0001
Clavien Dindo complications ^b^ (%)	21.9 *	20.0 *	18.3	<0.0001	64.7	<0.0001	18.1	0.0002	<0.0001
Transfer to intensive care unit (%)	22.8	22 ^‡^	23.3	0.0717	58.8	<0.0001	23.1	0.0483	<0.0001
In-hospital mortality(%)	2.6 *	2	2.1	0.0024	21.6	<0.0001	2	0.7079	<0.0001

^a^: Having at least one of the following complications during the surgery stay or within the first 30 days after the operation: pneumonia, acute respiratory distress syndrome, respiratory failure, heart failure, acute renal failure, infectious complications, pulmonary embolism. ^b^: Severe complications classified as grade III and IV in the Clavien Dindo classification (admission to ICU for more than 48 h, surgical revision or dialysis); * significantly more frequent compared to 2020 (*p* < 0.05); ^‡^ significantly less frequent compared to 2020 (*p* < 0.05).

**Table 3:** Logistic regression to study the effect of SARS-CoV-2 on the risk of 30-day in-hospital mortality and severe complications among lung cancer resection patients.

Characteristics	In-hospital Mortality		Severe Complications *	
	Crude OR (95% CI)	Adjusted OR (95% CI)	Crude OR (95% CI)	Adjusted OR (95% CI)
Year (reference = 2019, non-SARS-CoV-2)				
2020 without SARS-CoV-2 ^a^	1.050 (0.862–1.281)	1.097 (0.892–1.349)	0.908 (0.853–0.967)	0.904 (0.842–0.970)
2020 with SARS-CoV-2 ^b^	13.431 (6.793–26.555)	7.166 (3.302–15.552)	7.279 (3.872–13.684)	4.757 (2.309–9.799)
Type of lung cancer resection (ref = limited resection)				
Bilobectomy	3.479 (2.266–5.341)	2.356 (1.483–3.743)	2.345 (1.944–2.830)	1.923 (1.549–2.386)
Lobectomy	0.870 (0.649–1.165)	0.793 (0.586–1.072)	1.227 (1.118–1.346)	1.174 (1.059–1.302)
Pneumonectomy	3.700 (2.582–5.304)	2.939 (1.998–4.324)	2.470 (2.125–2.871)	2.145 (1.807–2.547)
Age	1.046 (1.034–1.058)	1.041 (1.028–1.054)	1.012 (1.009–1.015)	1.008 (1.004–1.012)
Men	2.509 (1.971–3.194)	1.670 (1.296–2.153)	1.632 (1.526–1.744)	1.320 (1.224–1.424)
Pulmonary disease	5.873 ((4.725–7.301)	4.091 (3.257–5.138)	6.709 (6.263–7.187)	5.911 (5.504–6.348)
Heart disease	3.090 (2.518–3.791)	1.496 (1.191–1.881)	2.484 (2.296–2.688)	1.690 (1.542–1.852)
Peripheral vascular disease	3.079 (2.437–3.890)	1.706 (1.323–2.202)	2.393 (2.170–2.640)	1.670 (1.491–1.871)
Neurological disease	3.043 (2.227–4.157)	1.846 (1.314–2.594)	1.766 (1.531–2.038)	1.261 (1.069–1.489)
Liver disease	7.398 (4.672–11.713)	4.990 (2.949–8.445)	2.291 (1.688–3.110)	1.727 (1.207–2.469)
Renal disease	4.882 (3.567–6.681)	2.581 (1.819–3.662)	2.252 (1.899–2.671)	1.509 (1.237–1.839)
Endocrine disease	1.706 (1.331–2.185)	0.978 (0.745–1.282)	1.359 (1.242–1.488)	1.033 (0.930–1.148)
Metabolic disease	2.830 (2.283–3.508)	1.543 (1.220–1.952)	2.196 (2.018–2.389)	1.437 (1.303–1.584)
Infectious disease	12.223 (7.864–18.999)	5.128 (3.082–8.532)	23.997 (13.526–42.574)	16.675 (9.056–30.704)
Hematological disease	3.091 (2.329–4.103)	1.386 (1.010–1.901)	2.381 (2.099–2.701)	1.407 (1.213–1.632)
Other malignant lesions	2.059 (1.688–2.512)	1.410 (1.141–1.742)	1.493 (1.402–1.590)	1.239 (1.153–1.331)
Other therapies **	1.848 (1.414–2.415)	-	1.603 (1.451–1.772)	-
Charlson index (reference = 0) **				
1	2.156 (1.611–2.884)	-	1.706 (1.573–1.851)	-
2	3.748 (2.665–5.271)	-	2.448 (2.182–2.747)	-
≥3	3.734 (2.87–4.851)	-	2.094 (1.933–2.267)	-

OR: odds ratio; CI: confidence interval. ^a,b^: Logistic regression in these analyses uses 2019 LC resection patients, who are obviously non-SARS-CoV-2 patients. * Having at least one of the following complications during the surgery stay or within the first 30 days after the operation: pneumonia, acute respiratory distress syndrome, respiratory failure, heart failure, acute renal failure, infectious complications, pulmonary embolism. ** Correlated with other variables and therefore not included in the multivariate model.

## Data Availability

The PMSI database was transmitted by the national agency for the management of hospitalization data. The use of these data by our department was approved by the national committee for data protection. We are not allowed to transmit these data. PMSI data are available for researchers who meet the criteria for access to these French confidential data (this access is submitted to the approval of the national committee for data protection) from the national agency for the management of hospitalization (ATIH—Agence technique de l’information sur l’hospitalisation). Address: Agence technique de l’information sur l’hospitalisation, 117 boulevard Marius Vivier Merle, 69329 Lyon CEDEX 03.

## Supplementary Materials

The following are available online at https://www.mdpi.com/article/10.3390/cancers13246277/s1, Figure S1: Frequency of patients who underwent pulmonary resection among all patients (*N* = 100,000 per year) hospitalized with a lung cancer code (main or associated diagnosis) by month, Figure S2: Frequency of patients who underwent pulmonary resection among all patients (*N* = 100,000 per year) hospitalized with a lung cancer code (main or associated diagnosis) by month and type of surgery, Table S1: Logistic regression to study the effect of SARS-CoV-2 on the risk of 30-day severe complications (with or without using Clavien Dindo classification) among lung cancer resection patients, Table S2: Logistic regression to study the effect of SARS-CoV-2 on the risk of 30-day in-hospital mortality and severe complications among lung cancer resection patients in 2020.
