# Supplementary material for: Impact of the SARS-CoV-2 Epidemic on Lung Cancer Surgery in France: A Nationwide Study
Source: Cancers (Basel). 2021 Dec 14;13(24):6277. doi: 10.3390/cancers13246277 (PMC8699699; doi:10.3390/cancers13246277)
Supplement: Supplementary file 1 [file cancers-13-06277-s001.zip › cancers-1423163-supplementary.pdf]

cancers

Supplementary Material: Impact of the SARS-CoV-2 Epidemic on Lung Cancer Surgery in France: A Nationwide Study

Pierre-Benoit Pages, Jonathan Cottenet, Philippe Bonniaud, Pascale Tubert-Bitter, Lionel Piroth, Jacques Cadranel, Alain Bernard and Catherine Quantin

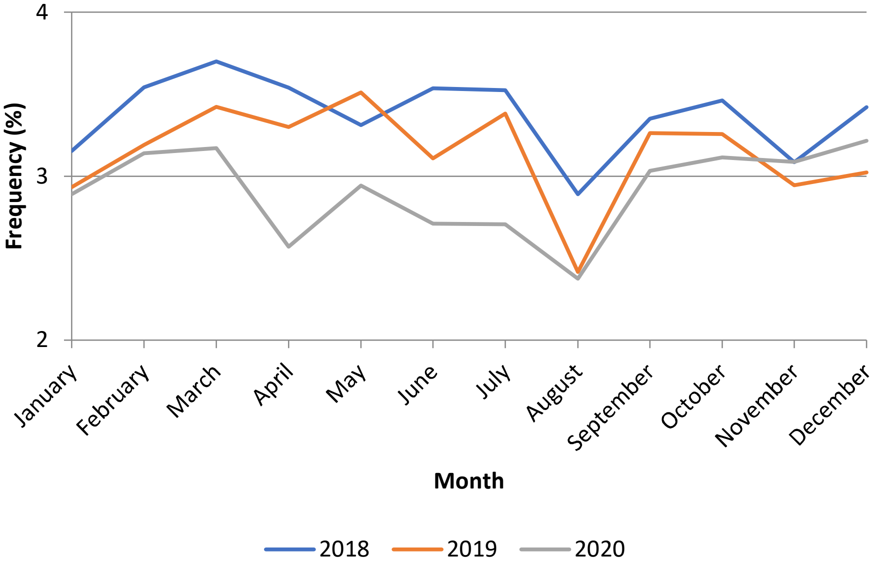

Figure S1. Frequency of patients who underwent pulmonary resection among all patients (N = 100,000 per year) hospitalized with a lung cancer code (main or associated diagnosis) by month.

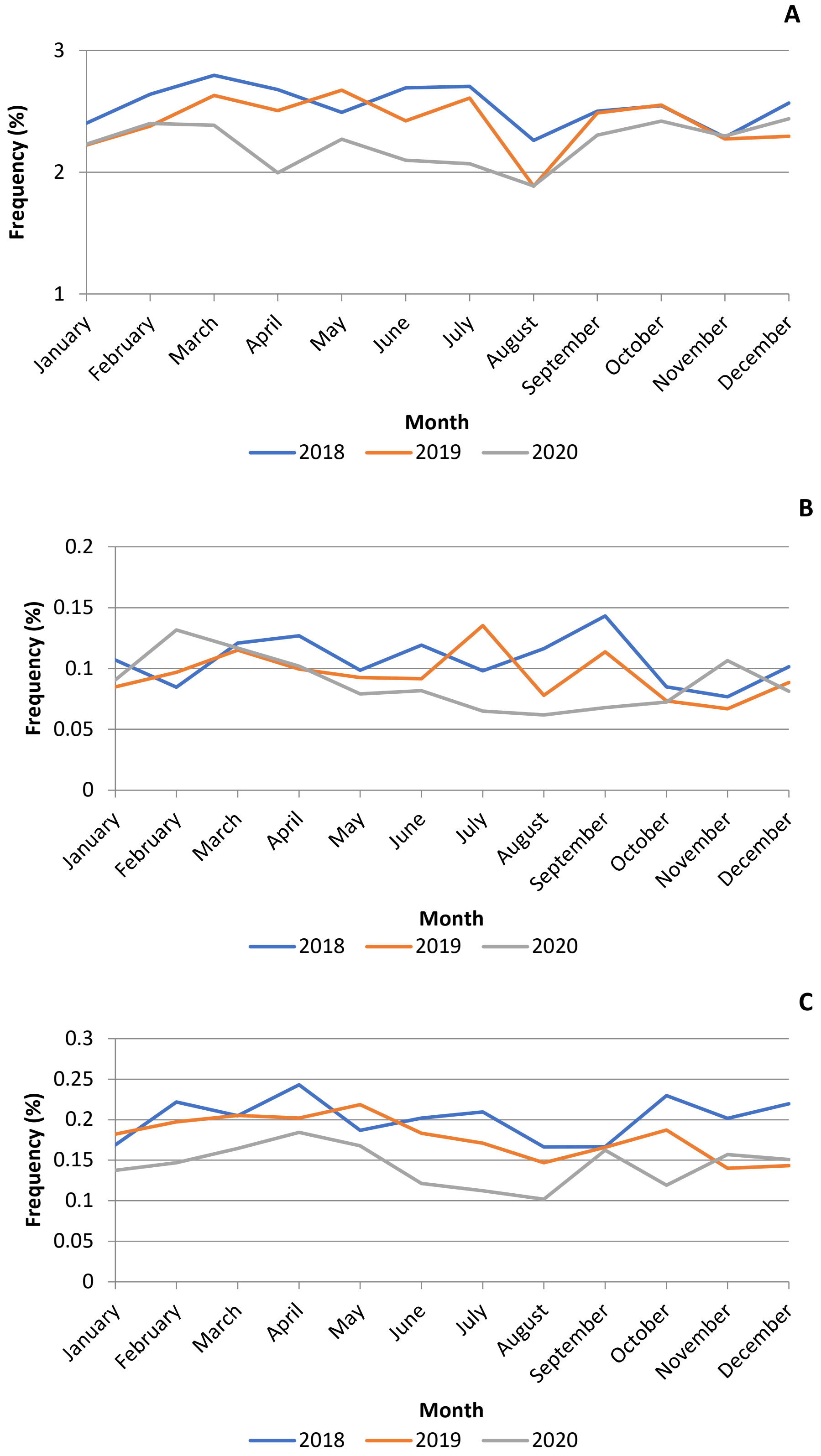

Figure S2. Frequency of patients who underwent pulmonary resection among all patients (*N* = 100,000 per year) hospitalized with a lung cancer code (main or associated diagnosis) by month and type of surgery. (**A**) Lobectomy; (**B**) Bilobectomy; (**C**) Pneumonectomy.

Table S1. Logistic regression to study the effect of SARS-CoV-2 on the risk of 30-day severe complications (with or without using Clavien Dindo classification) among lung cancer resection patients.

**Characteristics**	**Severe complications ***	**Severe complication using Clavien Dindo classification ****
	**Adjusted OR [95% CI]**	**Adjusted OR [95% CI]**
Year (ref = 2019, non-SARS-CoV-2)		
2020 without SARS-COV-2 [a]	0.904 [0.842–0.970]	0.865 [0.800–0.935]
2020 with SARS-COV-2 [b]	4.757 [2.309–9.799]	4.960 [2.5959–9.480]
Type of lung cancer resection (ref = limited resection)		
Bilobectomy	1.923 [1.549–2.386]	2.395 [1.9059–3.011]
Lobectomy	1.174 [1.059–1.302]	1.417 [1.255–1.601]
Pneumonectomy	2.145 [1.807–2.547]	2.795 [2.326–3.359]
Age	1.008 [1.004–1.012]	1.007 [1.002–1.011]
Men	1.320 [1.224–1.424]	1.479 [1.357–1.612]
Pulmonary disease	5.911 [5.504–6.348]	4.145 [3.830–4.485]
Heart disease	1.690 [1.542–1.852]	1.348 [1.221–1.487]
Peripheral vascular disease	1.670 [1.491–1.871]	1.571 [1.396–1.7681]
Neurological disease	1.261 [1.069–1.489]	1.401 [1.179–1.664]
Liver disease	1.727 [1.207–2.469]	2.207 [1.549–3.144]
Renal disease	1.509 [1.237–1.839]	1.500 [1.222–1.840]
Endocrine disease	1.033 [0.930–1.148]	0.981 [0.874–1.101]
Metabolic disease	1.437 [1.303–1.584]	1.390 [1.254–1.542]
Infectious disease	16.675 [9.056–30.704]	26.347 [14.676–47.300]
Hematological disease	1.407 [1.213–1.632]	1.529 [1.316–1.777]
Other malignant lesions	1.239 [1.153–1.331]	1.319 [1.219–1.427]

OR: odds ratio; CI: confidence interval, * having at least one of the following complications during the surgery stay or within the first 30 days after the operation: pneumonia, acute respiratory distress syndrome , respiratory failure, heart failure, acute renal failure, infectious complications, pulmonary embolism, ** severe complications classified as grade III and IV in the Clavien Dindo classification (admission to ICU for more than 48 hours, surgical revision or dialysis).

Table S2. Logistic regression to study the effect of SARS-CoV-2 on the risk of 30-day in-hospital mortality and severe complications among lung cancer resection patients in 2020.

**Characteristics**	**In-hospital mortality**	**Severe complications ***
	Adjusted OR [95% CI]	Adjusted OR [95% CI]
Year (ref = 2020, non-SARS-CoV-2)		
2020 with SARS-COV-2	6.883 [3.168–14.985]	5.238 [2.527–10.858]
Type of lung cancer resection (ref = limited resection)		
Bilobectomy	2.055 [1.014–4.165]	1.647 [1.198–2.264]
Lobectomy	0.848 [0.548–1.311]	1.165 [1.004–1.353]
Pneumonectomy	3.634 [2.114–6.245]	2.242 [1.739–2.890]
Age	1.034 [1.016–1.052]	1.011 [1.005–1.017]
Men	1.599 [1.121–2.280]	1.340 [1.199–1.497]
Pulmonary disease	3.843 [2.796–5.282]	6.253 [5.634–6.940]
Heart disease	1.691 [1.232–2.321]	1.700 [1.490–1.940]
Peripheral vascular disease	1.504 [1.038–2.180]	1.655 [1.401–1.955]
Neurological disease	1.436 [0.873–2.363]	1.326 [1.047–1.680]
Liver disease	3.722 [1.462–9.475]	1.434 [0.830–2.479]
Renal disease	2.469 [1.503–4.056]	1.341 [1.012–1.776]
Endocrine disease	1.362 [0.953–1.945]	0.939 [0.803–1.097]
Metabolic disease	1.291 [0.918–1.815]	1.548 [1.343–1.785]
Infectious disease	4.712 [2.218–10.011]	13.643 [5.810–32.039]
Hematological disease	1.554 [1.006–2.399]	1.397 [1.123–1.737]
Other malignant lesions	1.232 [0.917–1.656]	1.197 [1.078–1.330]

OR: odds ratio; CI: confidence interval, * having at least one of the following complications during the surgery stay or within the first 30 days after the operation: pneumonia, acute respiratory.
